# Inhibition of phosphatidylinositol 3-kinase by PX-866 suppresses temozolomide-induced autophagy and promotes apoptosis in glioblastoma cells

**DOI:** 10.1186/s10020-019-0116-z

**Published:** 2019-11-14

**Authors:** Bryan G. Harder, Sen Peng, Christopher P. Sereduk, Andrej M. Sodoma, Gaspar J. Kitange, Joseph C. Loftus, Jann N. Sarkaria, Nhan L. Tran

**Affiliations:** 1 Departments of Cancer Biology and Neurosurgery, Mayo Clinic Arizona, 13400 E. Shea Blvd, MCCRB 03-055, Scottsdale, AZ 85259 USA; 2 Cancer and Cell Biology Division, Translational Genomics Research Institute, Phoenix, AZ USA; 3 Department of Radiation Oncology, Mayo Clinic, Rochester, MN USA; 4 Department of Biochemistry and Molecular Biology, Mayo Clinic Arizona, Scottsdale, AZ USA

**Keywords:** Glioblastoma (GBM), Autophagy, Temozolomide (TMZ), PX-866, PI3K, Bafilomycin

## Abstract

**Background:**

Temozolomide (TMZ) is the most commonly used chemotherapeutic agent used to treat glioblastoma (GBM), which causes significant DNA damage to highly proliferative cells. Our observations have added to accumulating evidence that TMZ induces stress-responsive cellular programs known to promote cell survival, including autophagy. As such, targeting these survival pathways may represent new vulnerabilities of GBM after treatment with TMZ.

**Methods:**

Using the T98G human glioma cell line, we assessed the molecular signaling associated with TMZ treatment, the cellular consequences of using the pan-PI3K inhibitor PX-866, and performed clonogenic assays to determine the effect sequential treatment of TMZ and PX-866 had on colony formation. Additionally, we also use subcutaneous GBM patient derived xenograft (PDX) tumors to show relative LC3 protein expression and correlations between survival pathways and molecular markers which dictate clinical responsiveness to TMZ.

**Results:**

Here, we report that TMZ can induce autophagic flux in T98G glioma cells. GBM patient-derived xenograft (PDX) tumors treated with TMZ also display an increase in the autophagosome marker LC3 II. Additionally, O^6^-methylguanine-DNA-methyltransferase (MGMT) expression correlates with PI3K/AKT activity, suggesting that patients with inherent resistance to TMZ (MGMT-high) would benefit from PI3K/AKT inhibitors in addition to TMZ. Accordingly, we have identified that the blood-brain barrier (BBB) penetrant pan-PI3K inhibitor, PX-866, is an early-stage inhibitor of autophagic flux, while maintaining its ability to inhibit PI3K/AKT signaling in glioma cells. Lastly, due to the induction of autophagic flux by TMZ, we provide evidence for sequential treatment of TMZ followed by PX-866, rather than combined co-treatment, as a means to shut down autophagy-induced survival in GBM cells and to enhance apoptosis.

**Conclusions:**

The understanding of how TMZ induces survival pathways, such as autophagy, may offer new therapeutic vulnerabilities and opportunities to use sequential inhibition of alternate pro-survival pathways that regulate autophagy. As such, identification of additional ways to inhibit TMZ-induced autophagy could enhance the efficacy of TMZ.

## Introduction

Glioblastoma (GBM) is the most common and deadly primary adult brain tumor in adults with a median survival time of about 15 months from diagnosis (Thomas et al. 2014; Siegel et al. 2018). Numerous factors such tumor heterogeneity, invasiveness, and the blood-brain barrier (BBB) make treating this disease extremely challenging without compromising healthy brain tissue (Sottoriva et al. 2013; Patel et al. 2014; Xie et al. 2014; van Tellingen et al. 2015). Currently, investigations are underway to identify new therapeutics or novel ways to deliver drugs to the tumor site, but there have been minimal advancements since the implementation of the Stupp protocol in 2005, which only provides a moderate benefit on overall survival (Stupp et al. 2005). Therefore, an emphasis has been placed on identifying mechanisms to enhance the efficacy of temozolomide (TMZ), an orally administered alkylating agent, or radiation therapy (RT) to prolong patient survival.

Cells at the rim of a GBM are highly invasive and migratory which prevents complete surgical resection of tumors prior to standard therapy (Xie et al. 2014). The remaining cells are then targeted with RT and concomitant TMZ regimens. Both RT and TMZ are highly effective DNA-damaging agents, but recent work has shown that the remaining migratory cells display a certain degree of resistance to these therapies. Ultimately, the cells that survive this treatment regimen form a recurrent tumor. Currently, there are no effective treatment options for recurrent GBM that prolong overall survival and patients typically succumb to the disease after approximately 8 months (Roy et al. 2015).

Emerging evidence has begun to shed light on additional consequences of TMZ treatment. Notably, TMZ-induced methylation of DNA induces the DNA-damage response (DDR), which has recently been implicated to be associated with the induction of cell stress pathways such as macroautophagy (henceforth ‘autophagy’) (Hombach-Klonisch et al. 2018). Autophagy is a major catabolic process that is responsible for degrading cellular cargo too large or abundant for the proteasome (Levine and Kroemer 2008; Yin et al. 2016). Cargo targeted for degradation, such as misfolded proteins, protein aggregates, and damaged organelles are sequestered into forming autophagosome structures. These double-membrane autophagosomes containing cargo then fuse with lysosomes to create autolysosomes, where degradation takes place. After degradation of the internal components and completion of autophagic flux, the autolysosome breaks down and the resulting cellular constituents are released into the cytosol to be recycled by anabolic processes.

Cancer cells from multiple tumor types have been shown to have high basal autophagy, which is used as a cell-survival mechanism (White 2015; Degenhardt et al. 2006; Hu et al. 2012). When cells are deprived of nutrients, growth factor-induced signaling from receptor tyrosine kinases in the membrane is shut off. The PI3K/AKT/mTOR axis is a central signaling pathway that is activated as a result of growth factor stimulation and activation of class I PI3K functions as a negative regulator of autophagy (Nazio et al. 2013). When the PI3K/AKT/mTOR axis is not active, there is release of the inhibition on autophagic formation and autophagosome biogenesis begins through a signaling cascade that begins with the ULK1 complex (Russell et al. 2013). As a result of limited nutrients, autophagy is initiated, thus providing a growth advantage to tumor cells. However, there are some reports that showed that the class III PI3K Vps34(PIK3C3) forms a protein complex with BECN1 and PIK3R4 to generate phosphatidylinositol 3-phosphate, which is required for the initiation and progression of autophagy (Yu et al. 2015). In general, dual inhibitors of PI3K/mTOR are considered autophagy activators as both class I PI3K and mTOR (both of which suppresses autophagy) are simultaneously inhibited (Kuger et al. 2014). However, there is evidence that some of these dual inhibitors such as dactolisib and PI-103 can suppress autophagic flux in cancer cells (Button et al. 2016) via inhibition of class III PI3K. Moreover, autophagy is also a stress-responsive pathway, and cellular insult from chemotherapy also has shown to activate this process (Han et al. 2011; Ding et al. 2011; Paillas et al. 2012; Zou et al. 2012).

Previous studies have identified that the PI3K/AKT/mTOR axis is overactive in GBM and has been linked with enhanced proliferation, chemoresistance, and suppressed apoptosis (Parsons et al. 2008; Cheng et al. 2009). As a result, PI3K inhibitors have shown promising pre-clinical potential against GBM, however, none have achieved matriculation into the clinic (Zhao et al. 2017). Moreover, identification of compounds that can sensitize glioma cells to the effects of TMZ would be of major interest in the clinic. Here, we provide evidence that TMZ induces autophagic flux, and identify PX-866, an analog of wortmannin, as an early-stage blocker of autophagosome formation. Given these findings, we argue for the combined use of PX-866 to block TMZ-induced autophagy and augment GBM cell death.

## Materials and methods

### Cell culture

Human T98G glioma cells were purchased from the American Type Culture Collection (ATCC) and were maintained in Dulbecco’s Modified Essential Medium (DMEM) supplemented with 10% fetal bovine serum (FBS). U373vIII cells were maintained in DMEM supplemented with 10% tetracycline-free FBS and have been described previously (Roos et al. 2018). All cells were grown at 37 °C with 5% CO_2_.

### Generation of TMZ-resistant GBM PDX tumors

Parental GBM PDX lines were derived from either primary or recurrent tumor resections and have been described previously (Kitange et al. 2012). A comprehensive description of these parental GBM PDX models can be found at the (Mayo Clinic Brain PDX National Resource Portal n.d.). The TMZ-resistant lines were generated by treating subcutaneous parental GBM xenografts with escalating doses of TMZ (20 mg/kg/day X 3 days and then 66 mg/kg/day X 3 days after initial tumor re-growth). The resulting TMZ-resistant lines were completely resistant to a challenged 120 mg/kg/day X 5 days as described previously (Kitange et al. 2012).

### Western blot analysis

For experiments involving western blot analysis, cells were seeded in 6-well plates and left to adhere overnight. Cells were then treated with the indicated drugs and corresponding vehicle as a control for the appropriate time points. At the time of lysis, cells were washed once with ice-cold PBS containing protease and phosphatase inhibitor (Thermo scientific) cocktails. Cells were then lysed with 1x radioimmunoprecipitation assay (RIPA) buffer containing 1 mM phenylmethylsulfonyl fluoride (PMSF). Lysates were sonicated, and the total protein concentration was determined using the BCA assay kit, using bovine serum albumin as a standard. Equal protein amounts were loaded per well and resolved using SDS-PAGE. Protein was transferred to a nitrocellulose membrane using the Biorad fast transfer system, followed by blocking with Odyssey Blocking Buffer (LI-COR Biosciences) for 1 hour. The Odyssey CLx Near-Infrared (NIR) Western Blot Detection System (LI-COR Biosciences) was used to detect protein bands. Densitometry analysis was performed using the Image Studio Lite Version 5.2 software.

### Antibodies and reagents

The following primary antibodies purchased from Cell Signaling Technology (CST) include: PARP (9542), MGMT (2739), phospho-AKT (ser473) (4060), AKT (2920), and GAPDH (5174). The antibody against LC3 was purchased from Sigma (L7543), the antibody against p62 was purchased from Abnova [(M01), clone 2C11], and the antibody against α-Tubulin was purchased from Millipore. Primary antibodies were diluted in Odyssey Blocking Buffer with 0.2% Tween 20 and incubated overnight with gentle shaking at 4 °C. IRDye 800CW secondary antibodies (LI-COR Biosciences) were also diluted in Odyssey Blocking Buffer with 0.2% Tween 20 and incubated for 1 h at room temp with gentle shaking.

### Analysis of correlation between MGMT methylation status and PI3K-AKT activity

Patients included in this analysis were limited to the provisional TCGA microarray dataset with MGMT clinical status availability. Primary GBM were selected by filtering the “Sample Type” column in the patient clinical information table. Among the GBM microarray dataset, 190 MGMT unmethylated and 156 MGMT methylated samples were identified. Gene expression data is presented as normalized z-scores, which reflect the number of standard deviations away from the mean expression in the reference population. Gene set variation analysis (GSVA) was then performed to determine gene set enrichment scores of PI3K-AKT signaling pathway (Hanzelmann et al. 2013). The PI3K-AKT gene set was downloaded from Molecular Signatures Database (MSigDB) and modified to include additional canonical targets (Liberzon et al. 2015; Catasus et al. 2010). Log transformation was applied and default parameter settings were followed for gene expression data in GSVA. Student’s t-test was used to test the statistical significance of pathway activity difference between MGMT unmethylated and methylated groups. All figures were generated and statistical tests were performed using R 3.5.0 software. *P* values < 0.05 were considered significant.

### Assessment of cell viability

In order to determine the apoptotic state of cells after treatments involving TMZ, cleaved PARP was used as a marker. After completed treatment times, floating cells were collected from the media and adherent cells were harvested as mentioned above. Lysates from each treatment were combined and prepared for western analysis. Total cell lysate was resolved via SDS-PAGE and membranes were probed with a PARP antibody (CST) that recognizes both total and cleaved PARP.

### Colony formation assay

A colony formation (clonogenic) assay was used in order to determine cell survival after TMZ treatment either alone, or in combination with other treatments (Franken et al. 2006). Briefly, T98G or U373vIII cells were treated for 24 h with the indicated treatments. Remaining cells were trypsinized and plated in-triplicate into 6-well plates at a density of 1500 cells per well. Colonies were allowed to grow (approximately 9–10 days) before fixation with a 6% (v/v) glutaraldehyde (Sigma-Aldrich) solution. Fixed cells were then stained with a 0.5% (v/v) crystal violet (Sigma-Aldrich) solution and washed 3 times with de-ionized water. Only colonies consisting of ≥50 cells were counted. Statistical significance was determined using the Student’s *t* test and *P* values of < 0.05 were considered to be statistically significant.

## Results

### Temozolomide induces autophagic flux in glioma cells

**Fig. 1:**
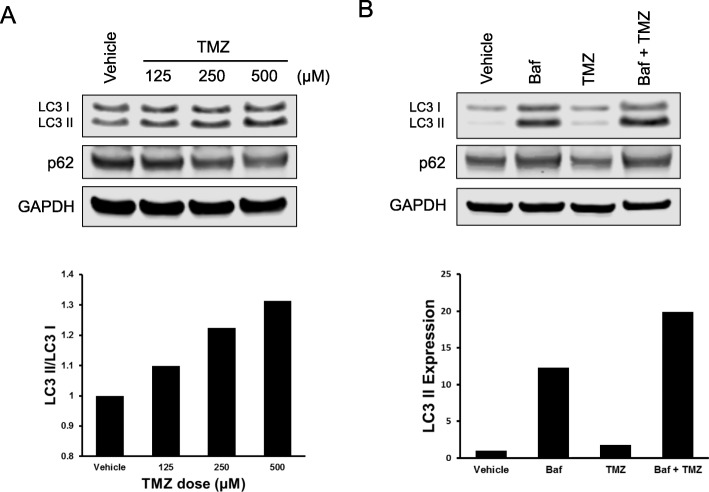
Temozolomide induces autophagic flux in glioma cells. **a** T98G cells were treated with increasing doses of TMZ for 48 h and only living (attached) cells were collected for analysis. Western blotting shows an increase in LC3 II protein levels and a decrease in p62 protein levels. GAPDH was used as a loading control. **b** The LC3 flux assay was performed on T98G cells. T98G cells were treated with vehicle or TMZ (500 μM) for 48 h containing either DMSO or Baf (10 nM) and only living (adherent) cells were collected. Western blotting for LC3 indicates an increase in LC3 II for the TMZ + Baf lane compared to Baf alone, proving TMZ-induced autophagosome formation. The data (LC3 II/GAPDH) was quantified and graphed to mark this increase. Data represents three independent experiments

Previous research has shown that treatment with TMZ has an effect on autophagy in various GBM cell lines. Our study used the T98G cell line to confirm the effect of TMZ on autophagy. Forty-eight hours treatment with TMZ induced a dose-dependent increase in LC3 II protein expression indicating that autophagy is being activated. To verify autophagy flux is induced by TMZ, we also measured the protein level of p62, a receptor for cargo destined to be degraded by autophagy, and itself is degraded upon autophagy induction. Upon TMZ treatment, p62 protein levels also degraded accordingly (Fig. 1a). To further confirm that the effect on autophagy is an induction rather than an inhibition of basal autophagy, the LC3 flux (turnover) assay was performed as previously reported (Mizushima et al. 2010). T98G cells were treated with vehicle or TMZ (500 μM) for 48 h, with or without the late-stage autophagy inhibitor Bafilomycin (Baf, 10 nM). Cells treated with TMZ and Baf showed a higher expression of LC3 II compared to the LC3 II expression of Baf treatment alone, confirming the production of autophagosomes by TMZ over the course of 48 h (Fig. 1b). Moreover, p62 levels decrease when treated with TMZ alone, but accumulate when treated with Baf or Baf plus TMZ, confirming that p62 is not being degraded because of an inhibition of autophagy completion.

### TMZ-resistant GBM-PDX tumors display increased LC3 II expression

**Fig. 2:**
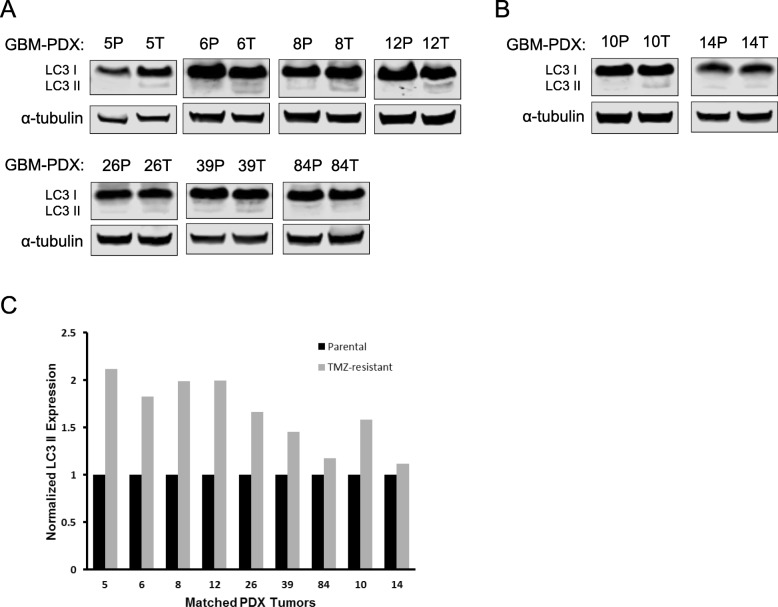
TMZ-resistant GBM-PDX tumors display increased LC3 II expression. Parental TMZ-sensitive (P) and TMZ-resistant (T) GBM-PDX subcutaneous tumors that were derived from either (**a**) primary tumor resections or (**b**) recurrent tumor resections were harvested and lysed for western blot analysis. LC3 protein levels were examined by western blotting and α-tubulin was used as a loading control. **c** Densitometry was used to quantify the western blot results

Next, we sought to assess the effect of TMZ on autophagy in vivo by utilizing subcutaneous GBM-PDX tumor samples from mice that received either vehicle or a dose-escalating regimen of TMZ. Lysate was created from tumors harvested from animals and western analysis was performed. Relative autophagosome number was determined by LC3 II expression between the parental TMZ-sensitive (P) and TMZ-resistant (T) groups for both primary (Fig. 2a) and recurrent (Fig. 2b) GBM-PDX tumor samples. In multiple GBM-PDX models, the TMZ-treated groups showed to have higher LC3-II expression compared to the parental groups, potentially indicating increased autophagosome number. Densitometry analysis revealed 6 out of 9 TMZ-treated tumors displayed a steady-state of 1.5-fold or higher increase in LC3 II expression (Fig. 2c).

### MGMT expression correlates with PI3K-AKT pathway activation

**Fig. 3:**
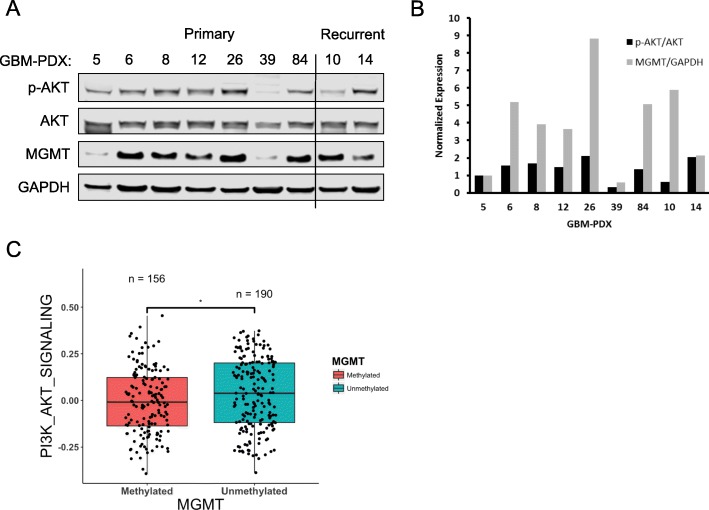
MGMT expression correlates with PI3K-AKT pathway activation. **a** Total protein from parental GBM-PDX subcutaneous tumor lysates was used to assess the differential expression of MGMT and relative activation of p-AKT between the samples. **b** Densitometry was used to quantify the western blot results and all PDX tumors were normalized to GBM5. **c** Gene set variation analysis (GSVA) was used to determine gene set enrichment scores of the PI3K-AKT signaling pathway according to the methylation status of the *MGMT* promoter in clinical samples

O^6^-methylguanine-methyltransferase (MGMT) is an important prognostic marker for patients with GBM (Hegi et al. 2005; Kitange et al. 2009). Because patients with high expression (low promoter methylation) of MGMT show resistance to TMZ, we examined if there was a correlation between MGMT expression and other pro-survival pathways in GBM clinical samples. Western blotting of GBM-PDX tumor lysate reveals differential expression of MGMT between tumor samples, which correlated with the activation of the PI3K-AKT axis across all primary-derived GBM-PDX tumor specimens, but not for recurrent GBM-PDX (GBM10) (Fig. 3a). Relative AKT activation and MGMT expression was correlated based on the GBM5 sample, which shows low p-AKT and low MGMT expression (Fig. 3b). We next characterized this correlation further by exploring the TCGA GBM cohort of large-scale patient samples with genomic and clinical profiles. Patient samples with available *MGMT* promoter methylation status and gene expression data were used to query PI3K-AKT pathway activity. Primary GBM patients with unmethylated *MGMT* have statistically significant higher PI3K-AKT activity compared to *MGMT* methylated cases (Fig. 3c).

### PX-866 blocks autophagy in glioma cells

**Fig. 4:**
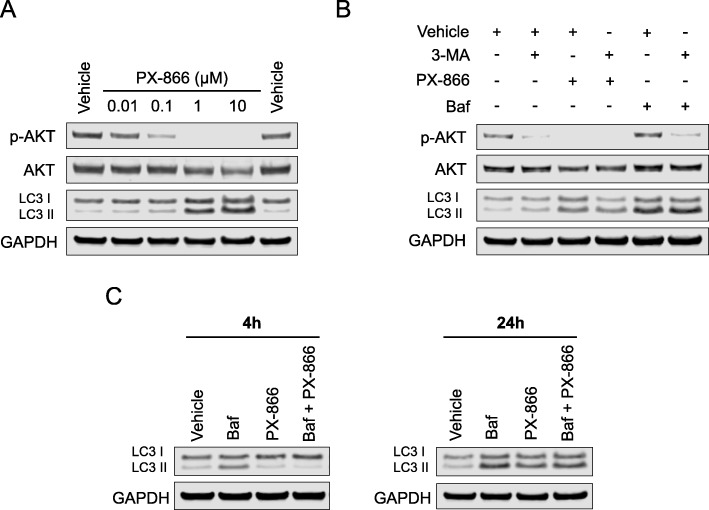
PX-866 blocks autophagy in glioma cells. **a** T98G cells were treated with increasing doses of PX-866 for 24 h and then total protein was isolated. **b** T98G cells were subjected to single treatment with 3-MA (2.5 mM) or co-treatment with either PX-866 (1 μM) or Baf (10 nM) for 24 h, according to the treatment legend. **c** The LC3 flux assay was performed on T98G cells at 4 h and 24 h using Baf (10 nM), PX-866 (1 μM) or a combination of the two. Data represents three independent experiments

Given that over-activation of the PI3K pathway is frequently reported in primary GBM, we decided to explore the signaling consequences of the pan-PI3K inhibitor PX-866, due to its previous use in the clinic for recurrent GBM. T98G cells treated with increasing doses of PX-866 dose-dependently inhibited the phosphorylation of AKT (ser473), but also increased the expression of both LC3 I and LC3 II, which is indicative of a late-stage autophagy blocker (Fig. 4a). In addition, p62 protein was increased with the treatment of PX-866 (Additional file 1: Figure S1). We then used 3-MA (PI3K inhibitor/early autophagy blocker) to determine if there was any functional similarity between PX-866 and Baf. Co-treatment of 3-MA with PX-866 was able to inhibit the accumulation of both LC3 I and II compared to PX-866 alone, while co-treatment of 3-MA with Baf showed no alteration in LC3 protein levels compared to Baf alone, indicating that PX-866 is not a late stage inhibitor like Baf (Fig. 4b). To further explore this mechanism, an LC3 flux assay was performed using early (4 h) and late (24 h) time points. At a 4 h time point, PX-866 alone and in combination with Baf was able to cause an accumulation of LC3 I and a reduction in LC3 II. At 24 h, PX-866 in combination with Baf showed a reduction of LC3 II levels compared to Baf alone (Fig. 4c). These data provide evidence that PX-866 is an early-stage inhibitor of autophagosome formation, thereby preventing the conversion of LC3 I to LC3 II.

### TMZ and PX-866 cooperate to diminish survival

**Fig. 5:**
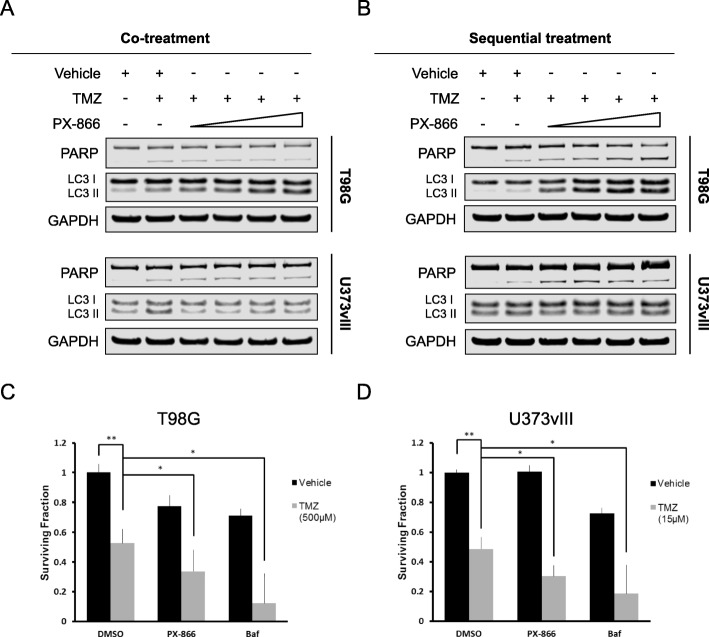
TMZ and PX-866 cooperate to diminish survival. **a** T98G (EGFR WT) and U373vIII (EGFRvIII) were treated with TMZ (500 μM) and increasing doses of PX-866 for 48 h (co-treatment). Both floating and remaining adherent cells were collected for analysis and western blotting for PARP and LC3 was used to assess apoptosis and autophagy, respectively. The triangle representing the dose escalation for PX-866 indicates: 0.125, 0.25, 0.5, and 1 μM. **b** T98G and U373vIII cells were treated with TMZ (500 μM) for 24 h, followed by the addition of PX-866 for another 24 h (sequential treatment). The triangle representing the dose escalation for PX-866 indicates: 0.125, 0.25, 0.5, and 1 μM. T98G cells (**c**) and U373vIII cells (**d**) were treated with the indicated treatments for 24 h. Cells were trypsinized 24 h after drug treatment and re-plated in 35 mm dishes in triplicate and allowed to form colonies. After about 9–10 days, colonies were fixed (6% glutaraldehyde) and stained (0.5% crystal violet). The number of colonies were counted and presented as a bar graph. Values are the mean ± the standard deviation of three separate measurements, *, *P* < 0.05 and, **, *P* < 0.01. Data represents the mean and S.D. from three independent experiments with each experiment conducted in triplicates

TMZ-induced autophagy could potentially facilitate GBM cell survival, but it may also represent a therapeutic vulnerability. It is well established that inhibition of autophagy with lysosomotropic compounds enhances the efficacy of conventional chemotherapy (Degtyarev et al. 2008; Apel et al. 2008; Buccarelli et al. 2018). Given that PX-866 inhibits autophagy, we set out to determine if the addition of PX-866 to TMZ could augment apoptosis in T98G cells. The addition of increasing doses of PX-866 to TMZ for 48 h had no effect on the cleavage of PARP, a traditional marker of apoptosis, compared to TMZ alone, but did show an induction of both LC3 I and LC3 II (Fig. 5a) and p62 (Additional file 1: Figure S1) protein levels, indicating a blockage of autophagy. This observation was confirmed in U373vIII cells, which have high activation of the PI3K pathway due to the presence of the EGFRvIII receptor which is constitutively active (Learn et al. 2004; Golding et al. 2009). Interestingly, treatment with TMZ first for 24 h, followed by the addition of PX-866 for another 24 h, induced the cleavage of PARP in a dose-dependent manner in both T98G and U373vIII cells, while consistently causing an induction of both LC3 I and LC3 II protein levels (Fig. 5b). Accordingly, both T98G and U373vIII cells exhibited a decrease in the ability to form colonies after co-treatment with TMZ (500 μM for T98G, and 15 μM for U373vIII) and PX-866 (both 1 μM) compared to TMZ alone (Fig. 5c and d). The addition of Baf to TMZ had a more pronounced effect on colony formation compared to TMZ and PX-866, potentially indicating that lysosomotropic inhibitors may be more effective due to their ability to inhibit the late stage of autophagy compared to inhibition of initiation.

## Discussion

In this report we have provided mechanistic evidence for the use of a new treatment strategy for GBM. TMZ was shown to induce autophagic flux and inhibition of TMZ-mediated autophagy with PX-866 increased apoptosis and significantly diminished the capacity of GBM cells to form colonies compared to TMZ alone.

TMZ has been the mainstay chemotherapy for GBM since 2005 and is effective against highly proliferative cells through alkylation of DNA. TMZ has been shown to activate the autophagy pathway, but there is little evidence to indicate how this occurs (Kanzawa et al. 2004; Wurstle et al. 2017). Induction of the DNA damage response (DDR) has recently been associated with the activation of autophagy and other cellular programs to mitigate stress and promote survival (Eliopoulos et al. 2016). Stress-responsive programs, such as the unfolded protein response (UPR), could play a role in the induction of autophagy, potentially through transcriptional activation of key autophagy genes (Ogata et al. 2006).

The autophagy inhibitor chloroquine (CQ) has shown pre-clinical promise as a chemosensitizing agent which can enhance the effects of TMZ (Lee et al. 2015; Hori et al. 2015; Golden et al. 2014). The addition of CQ to radiation and TMZ was shown to be clinically effective for newly diagnosed GBM compared to standard care alone, but the efficacy of this combination was restricted by dose-limiting toxicity associated with CQ (Briceno et al. 2007; Rosenfeld et al. 2014). In this study, we have provided evidence that PX-866 blocks autophagosome maturation in T98G cells, identifying it as an inhibitor of autophagy. PX-866, an irreversible pan-PI3K inhibitor, is able to penetrate the BBB and has previously been shown to minimize tumor growth in a flank xenograft model of GBM in mice (Koul et al. 2010). It has also been reported that PX-866 does not induce apoptosis in GBM cells, but significantly reduces the expression of VEGF and the invasion of GBM cells in vitro (Koul et al. 2010).

Multiple genomic alterations confer overactive PI3K-AKT-mTOR signaling in GBM. Amplification of *EGFR*, the expression of EGFRvIII, loss of *PTEN*, and mutations in *PIK3CA* represents a large portion of driver mutations in GBM which converge on the AKT-mTOR signaling axis (Prados et al. 2015; Brennan et al. 2013). Despite a plethora of signaling effectors for potential therapeutic intervention, no compounds targeting this pathway have made it into the clinic for the treatment of GBM. A phase II clinical trial was performed with PX-866 in patients with recurrent GBM (Pitz et al. 2015). Used as a monotherapy, PX-866 did not offer an advantage in overall survival. Other molecules, such as BEZ235, a dual PI3K and mTOR inhibitor, has shown promising pre-clinical evidence against GBM, however, BEZ235 has poor BBB-penetrance and is a strong autophagic inducer, potentially allowing cells to achieve autophagy-dependent survival.

There is evidence to support the fact that autophagic induction promotes GBM cell survival and may be a critical mechanism of adaptive chemoresistance (Fan et al. 2010; Fan and Weiss 2011). The majority of drugs targeting the PI3K/AKT/mTOR axis inhibit class 1 PI3K, which promotes autophagy due to the inhibitory effect on mTOR. Interestingly, our findings using PX-866 showed a blocking effect on autophagy, potentially through inhibition of class III PI3K (VPS34) which helps to control autophagosome biogenesis (Russell et al. 2013; Stjepanovic et al. 2017). Understanding the expression and activity of the different types of PI3K enzymes present in certain GBM cell lines or PDX models may help to ultimately determine the overall effect certain inhibitors may have on autophagy. As such, this potentially may lead to the identification of therapeutic vulnerabilities in GBM and provide a translatable approach to create rational drug combinations to treat GBM (Jutten et al. 2018).

Patients with newly diagnosed GBM will invariably receive the standard care of radiation therapy and TMZ after surgical removal of the tumor regardless of their expression status of MGMT. In cases where MGMT expression is high, these patients will respond poorly to TMZ, however, if we begin to understand additional features of tumors with inherent resistance to TMZ, this also may offer new therapeutic combinations to drugs that are readily available for repurposing efforts. The correlation between PI3K pathway activation and MGMT promoter methylation status may be an early insight into treating patients with inherent TMZ resistance with PI3K inhibitors that can inhibit autophagy or late-stage autophagy blockers such as CQ. Thus, these data provide mechanistic evidence that patients with high expression of MGMT and a high degree of PI3K activity may respond well to the use of TMZ followed by a pan-PI3K inhibitor/autophagy blocker added in a sequential manner.

## Conclusion

**Fig. 6:**
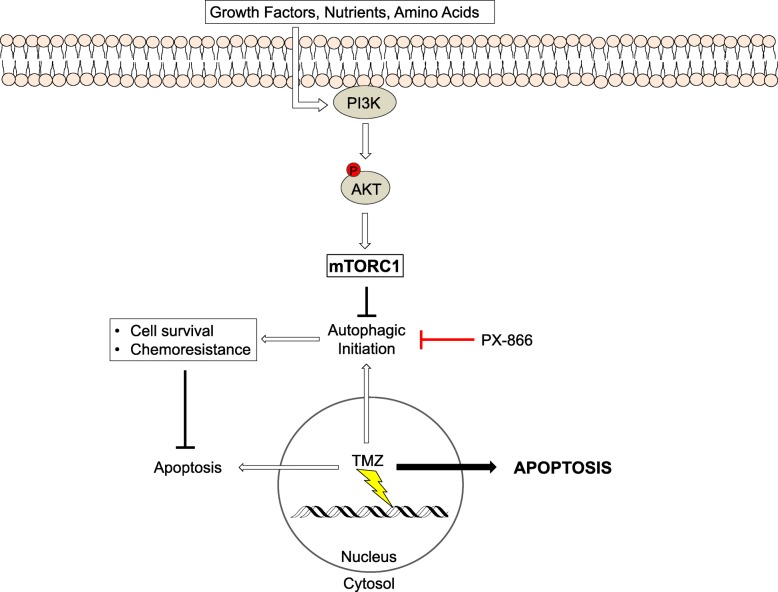
Schematic of the proposed mechanism of how PX-866 cooperates with TMZ to enhance apoptosis through inhibition of TMZ-induced autophagy

We have identified that treating with TMZ followed by the addition of the pan-PI3K inhibitor PX-866 promotes glioma cell death. We provide evidence that TMZ-induced autophagy is a delayed response, thus providing an opportunity to sequentially target this cell survival mechanism with PX-866 (Fig. 6). Understanding the correlation between MGMT status and relative activation of key survival pathways in GBM may provide therapeutically-relevant discernments for a more personalized approach towards treating newly diagnosed GBM in the future.

## Supplementary information

**Additional file 1: Figure S1.** T98G cells were treated with PX-866 or TMZ alone or in combination for 24 h and then total protein was isolated. p62 protein levels were examined by western blotting, and GAPDH was used as a loading control.

## Data Availability

Readily available upon request.

## Abbreviations

(3-MA: 3-Methyladenine
Baf: Bafilomycin
BBB: Blood-brain barrier
CQ: Chloroquine
DDR: DNA-damage response
EGFR: Epidermal growth factor receptor
GBM: Glioblastoma
GSVA: Gene set variation analysis
MGMT: O^6^-methylguanine-DNA- methyltransferase
MSigDB: Molecular Signatures Database
PARP: Poly (ADP-ribose) polymerase
PDX: Patient-derived xenograft
PI3K: Phosphatidylinositol 3-kinase
PTEN: Phosphatase and tensin homolog
RT: Radiation therapy
TMZ: Temozolomide
