# Supplementary material for: Inhibition of phosphatidylinositol 3-kinase by PX-866 suppresses temozolomide-induced autophagy and promotes apoptosis in glioblastoma cells
Source: Mol Med. 2019 Nov 14;25:49. doi: 10.1186/s10020-019-0116-z (PMC6854621; doi:10.1186/s10020-019-0116-z)
Supplement: Supplementary file 1 — Additional file 1: Figure S1. T98G cells were treated with PX-866 or TMZ alone or in combination for 24 h and then total protein was isolated. p62 protein levels were examined by western blotting, and GAPDH was used as a loading control. [file 10020_2019_116_MOESM1_ESM.pptx]

## Slide 1
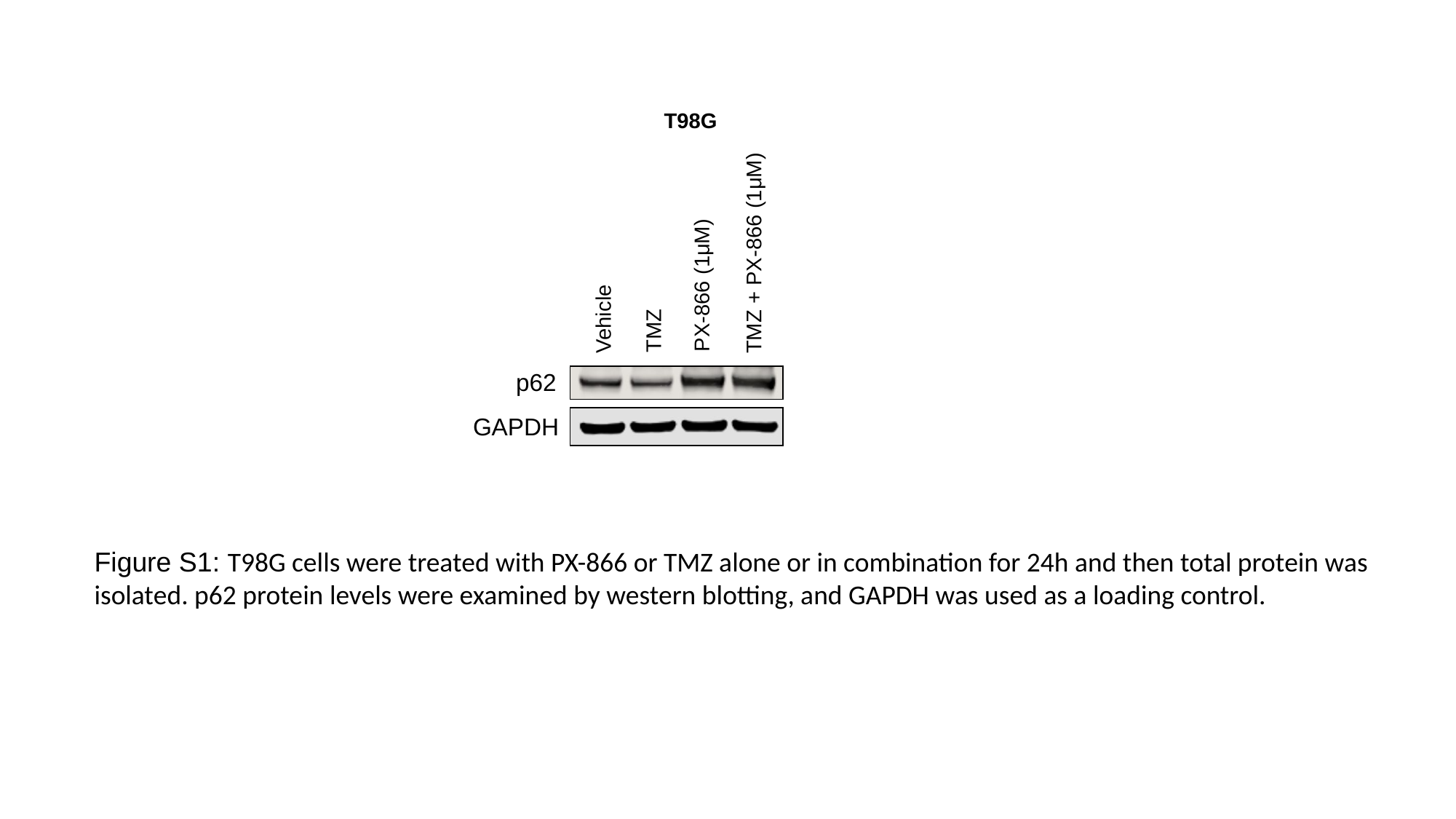

T98G
TMZ + PX-866 (1μM)
PX-866 (1μM)
Vehicle
TMZ
p62
GAPDH
Figure S1: T98G cells were treated with PX-866 or TMZ alone or in combination for 24h and then total protein was isolated. p62 protein levels were examined by western blotting, and GAPDH was used as a loading control.
